# The Role of Vasculature and Angiogenic Strategies in Bone Regeneration

**DOI:** 10.3390/biomimetics9020075

**Published:** 2024-01-26

**Authors:** Hye-Jeong Jang, Jeong-Kee Yoon

**Affiliations:** Department of Systems Biotechnology, Chung-Ang University, Anseong-si 17546, Gyeonggi-do, Republic of Korea; misodam0527@gmail.com

**Keywords:** angiogenesis, bone regeneration, osteogenesis, vasculature

## Abstract

Bone regeneration is a complex process that involves various growth factors, cell types, and extracellular matrix components. A crucial aspect of this process is the formation of a vascular network, which provides essential nutrients and oxygen and promotes osteogenesis by interacting with bone tissue. This review provides a comprehensive discussion of the critical role of vasculature in bone regeneration and the applications of angiogenic strategies, from conventional to cutting-edge methodologies. Recent research has shifted towards innovative bone tissue engineering strategies that integrate vascularized bone complexes, recognizing the significant role of vasculature in bone regeneration. The article begins by examining the role of angiogenesis in bone regeneration. It then introduces various in vitro and in vivo applications that have achieved accelerated bone regeneration through angiogenesis to highlight recent advances in bone tissue engineering. This review also identifies remaining challenges and outlines future directions for research in vascularized bone regeneration.

## 1. Introduction

Bones play a crucial role in the human body as they provide structural support, protect vital organs, and store essential minerals [1,2]. The process of bone regeneration involves the regrowth of osteocytes, as well as other functional cells such as endothelial cells (ECs) and neurons for revascularization and innervation, restoring the bone’s original shape and function [3,4,5]. These regenerative cascades are activated in response to bone defects, caused by traumatic injury, bone tumors, cysts, or infections, when the inherent adaptability of bone allows it to undergo spontaneous remodeling [6,7]. However, critical-size bone defects, which are clinically defined as defects with a length exceeding 1–2 cm in length and involving a loss of more than 50% of bone circumference, can cause non-healing fractures and reduced mobility, posing a significant challenge in clinical settings [8,9]. Inappropriate treatment of critical-size bone defects can exacerbate the condition, potentially causing malunion (abnormal or misaligned healing of the fractured bone ends) or nonunion (failure of the fractured bone ends to heal) [10,11,12]. These complications can result in persistent pain, deformity, impaired joint function, and, in some cases, limb shortening, adversely impacting the quality of life [13,14,15].

Conventional bone tissue engineering centered on autologous bone grafts is the gold standard for replacing fractured bone with the highest potential for healing [16,17]. However, the surgery required for bone harvest is repeatedly reported to result in sequential complications [18,19]. As an alternative, allogenic bone grafts offer improved safety, size and shape diversity, and reduced donor-site morbidity, as well as reducing the time required for graft preparation [20,21,22]. However, inadequate supply, risks of immune responses due to allograft rejection, and a decrease in osteoinductivity and osteoconductivity during storage, resulting from alterations in mechanical and biological properties, have been reported [23,24,25]. These disadvantages in autologous and allogeneic bone grafts highlight the need for artificial bone substitutes that closely resemble natural bone, particularly in terms of mechanical and biological properties, which can significantly impact the success rate of implants.

Artificial bone substitutes are composed of a diversity of various biomaterials, such as metals or bioceramics with precisely controlled structural properties, to regulate the biomechanical regenerative mechanism and enhance the osteoinductivity, osteoconductivity, and osseointegrity. Since bone is a highly vascularized tissue [26], a recent approach for bone regeneration involves treating bone defects by inducing angiogenesis in the bone complexes. Promoting the formation of blood vessels in new bone is essential for achieving high levels of biostability and biosafety, as it allows for the establishment of connections between newly generated vascular structures at the defect sites and the pre-existing blood vessels of the host, eventually nourishing the new bone and regulating immune responses [27]. Moreover, the angiogenic factors, including the vascular cells (e.g., ECs), growth factors (e.g., vascular endothelial growth factor; VEGF), or the hypoxic condition, are known to directly contribute to osteogenesis by stimulating mesenchymal stem cells (MSCs) and osteoblasts [28,29,30].

Here, we review the recent strategies for inducing angiogenesis to promote bone regeneration. Advances in tissue engineering technologies have enabled the coupling of osteogenesis and angiogenesis during bone regeneration through the use of mechanical and biological factors, including their mechanistic studies. From in vitro to in vivo applications, our review emphasizes the critical role of angiogenesis as a new strategy in designing artificial bone substitutes for successful bone regeneration, providing insights that span from controlled in vitro environments to the promising landscape of in vivo interventions.

## 2. Angiogenesis in Bone Regeneration

Bone regeneration occurs spontaneously in response to bone injury, involving multiple factors such as growth factors, osteoblasts, osteoclasts, inflammatory cytokines, and the extracellular matrix [31]. Since the bone microenvironment is a sophisticated system involving physiological, chemical, and physical factors, bone regeneration strategies utilize various aspects such as scaffolding [32], cell therapy [33], growth factor delivery [34], cytokine incorporation [35], immunomodulation [36], and angiogenesis [37] to target the microenvironment. The process of bone regeneration is a dynamic and organized procedure that typically consists of three phases: the inflammatory phase, the bone production phase, and the bone remodeling phase [38,39,40]. When a bone defect occurs, an acute inflammatory response follows bleeding, resulting in hematoma formation with a hypoxic, low pH, calcium-rich microenvironment [41,42]. This recruits inflammatory cells to the defect site and initiates the pro- and anti-inflammatory cascades, which further stimulate the angiogenic physiology [43]. During the bone production phase, the hematoma is replaced by soft tissue such as fibrous tissue or cartilage tissue (e.g., soft callus), which gradually hardens over several weeks [44,45]. MSCs differentiate into osteoprogenitor cells, and vascularization occurs in this phase [46]. Finally, the bone remodeling phase then restores the structural and mechanical properties, achieving a balance between osteoblastic and osteoclastic activity.

Bone fractures can cause damage to blood vessels and bleeding, leading to a hypoxic condition that can induce inflammation and hinder the regenerative process [47]. Therefore, angiogenesis, which involves endothelial sprouting from pre-existing blood vessels, is crucial for enhancing the regenerative capability during bone regeneration by salvaging the new bone tissue from hypoxia and supplying oxygen and nutrients through a consistent perfusion [48]. Moreover, a mechanism known as the coupling of osteogenesis and angiogenesis suggests that the neovasculature also stimulates the osteoprogenitor cells via angiogenic paracrine signaling, promoting their proliferation or differentiation [49,50].

**Figure 1:**
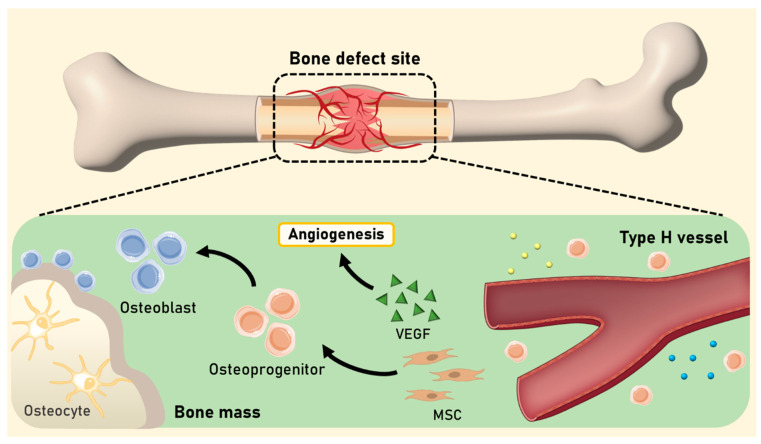
Schematic illustration of the mechanism of bone regeneration and angiogenesis within a bone defect site. Type H vessels, characterized by the expression of Endomucin and CD31, play an important role in the modulation of osteogenesis. Osteoprogenitor cells undergo sequential differentiation, transforming into osteoblasts and subsequently maturing into osteocytes. The interplay of VEGF secreted by endothelial cells within type H vessels, along with MSCs, serves as a potent inducer of angiogenesis and ultimately contributes to bone regeneration.

The precise mechanisms of bone vasculature formation and the osteogenic–angiogenic coupling still remain poorly understood. Several studies suggest that type H vessels, which express high levels of Endomucin and CD31 on the endothelium, modulate osteogenesis during the bone production to bone remodeling phase (Figure 1) [51,52]. Type H vessels are surrounded by osteoprogenitor cells and guide their proliferation and differentiation into osteocytes [53]. For instance, in endochondral ossification, Type H ECs secrete a high level of VEGF into the cartilage to induce angiogenesis, recruiting chondroclasts and osteoblasts to remodel the soft cartilage into hard bone tissue [54]. In addition, platelet-derived growth factor (PDGF) secreted from osteoclasts is known to recruit such ECs to induce bone formation [55]. In intramembranous ossification, a group of MSCs (i.e., mesenchymal condensation) secrete a high level of VEGF, attracting endothelial cells [56,57]. The ECs stimulate the MSCs to initiate osteogenic differentiation, leading to ossification and mineralization [58,59].

## 3. Conventional Strategies for Vascularized Bone Tissue Engineering

Current bone tissue engineering methods primarily rely on the use of autologous or allogenic bone grafts. These grafts exhibit good biomechanical properties, including an ideal elastic modulus similar to natural bone, as well as good osteoconductivity and osteoinductivity, making them suitable for replacing the bone defect. However, limitations such as poor availability and donor site morbidity in autologous bone grafts, as well as graft rejection in allogeneic bone grafts, may lead to implant failure and cause significant complications [59,60,61]. Therefore, the development of artificial bone substitutes using synthetic materials has emerged as a viable alternative. Metals such as titanium, stainless steel, or chromium alloys have been widely used due to their high mechanical strength, wear resistance, and durability [62,63]. However, the non-biodegradable nature of metal implants may require secondary surgery for removal, and their mechanical properties may not match those of the surrounding bone, leading to stress shielding and subsequent bone resorption [64,65,66,67]. Bioceramics, such as hydroxyapatite, tricalcium phosphate, or their mixture, known as biphasic calcium phosphate, are biocompatible and support bone ingrowth due to their osteoconductive properties, as they consist of a high amount of calcium and phosphate ions [68,69,70,71,72,73]. However, the brittleness and lower mechanical strength of ceramic implants compared to metals limit their use to non-load-bearing sites [74,75].

The material properties of artificial bone substitutes are recently being improved by synthesizing optimal materials using biodegradable polymers, or various composites of metal, ceramic, and polymeric materials with innovative techniques. However, the biological response, such as angiogenesis induction, has not been resolved. Conventional tissue engineering strategies have enabled vascularization within the implanted scaffolds by creating interconnected micro- or macroporous structures. When designing porous materials, the selection of an optimal pore size range is important to control the biomolecule diffusion, cell infiltration, and the behavior of the adhered cells. For example, smaller pores have been shown to induce cell differentiation, whereas larger pores induce cell proliferation [76,77]. In contrast, smaller pores may lead to a hypoxic condition within the implant due to low diffusion efficiency and stimulate chondrogenesis rather than osteogenesis [78,79].

In terms of vascularization, the natural hypoxic environment in the bone defect will stimulate MSCs to secrete proangiogenic factors that promote angiogenesis [80]. Typically, macropores with a size larger than 100 μm are preferred over micropores with a size smaller than 20 μm, since larger pores better induce the infiltration of ECs, resulting in a higher osseointegration of the implant [81,82]. However, the need for micropores should also be considered to modify the surface properties, such as capillarity, which can lead to a more homogeneous cell distribution and higher bone volume fraction. Therefore, scaffolds with a variety of pore sizes are currently being developed. Regarding porosity, a higher porosity level (>80%) facilitates better cell infiltration and higher interconnectivity but decreased mechanical properties, leading to material failure under high stress [78,83]. Overall, porous scaffolds can improve osseointegration through vascular infiltration [84]. However, spontaneous angiogenesis may not be fast enough to form a dense vascular network in a bone defect larger than a critical-size defect. Therefore, it is important to consider vascular engineering in addition to osteogenesis. This can be achieved by utilizing proangiogenic cells or growth factors, or through advanced material design (Table 1).

## 4. Angiogenic Strategies for Bone Regeneration

### 4.1. Angiogenic Factor Delivery

**Figure 2:**
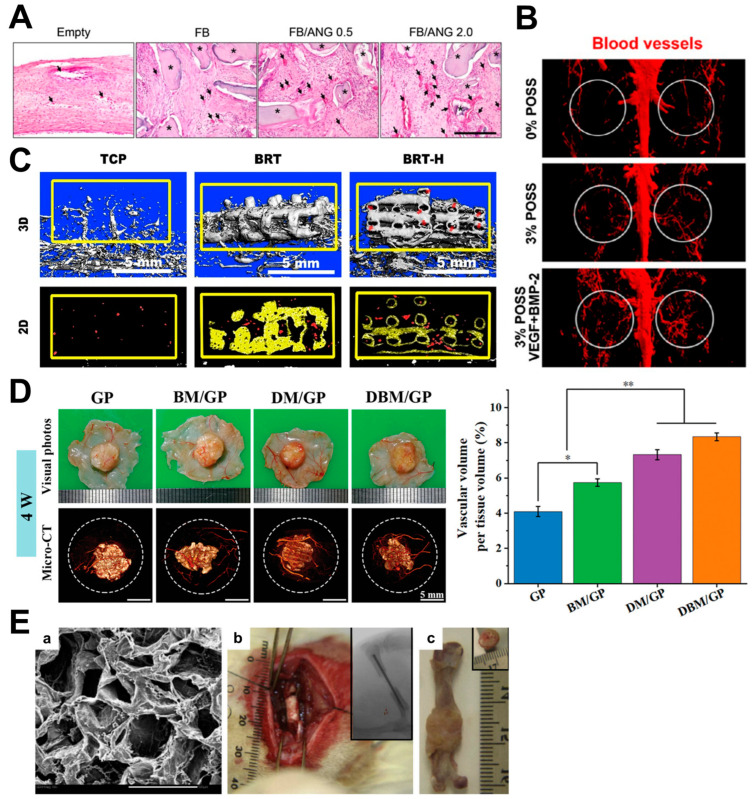
(**A**) Hematoxylin–Eosin staining image of enhanced vascularization at the defect site after implantation, showing an increased number of blood vessels [86]. Reprinted with permission from Kim et al., Copyright © 2024 Springer Nature. (**B**) MicroCT image showing the effects of POSS hydrogels on in vivo vascularization in the rat calvarial defect model with a higher number of blood vessels [87]. Reprinted with permission from Chen et al., Copyright © 2024 American Chemical Society. (**C**) MicroCT image showing enhanced vessel formation in the defect sites after BRT-H scaffold implantation in a rabbit radial defect model (red arrows) [89]. Reprinted with permission from Zhang et al., Copyright © 2024 Elsevier Ltd. (**D**) Digital images of harvested samples and microCT results to evaluate in vivo vascularization and bone regeneration in a rat calvarial defect model showing increased neovascularization after nanofibrous scaffold implantation [90]. Reprinted with permission from He et al., Copyright © 2024 John Wiley & Sons, Inc. (**E**) (a) PCL-HA composite and its (b) implantation surgery, as well as (c) observation of harvested implants showing bone integration and improved vascularization [93]. Reprinted with permission from Yu et al., Copyright © 2024 Elsevier Ltd.

**Figure 3:**
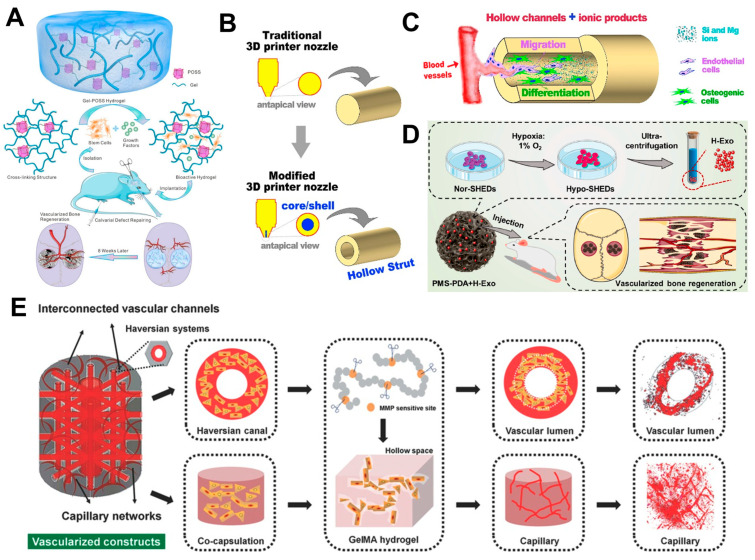
(**A**) Schematic illustration of structural design of POSS hydrogels and their application for in vivo bone defect repair [87]. Reprinted with permission from Chen et al., Copyright © 2024 American Chemical Society. (**B,C**) Schematic illustration of the printing and fabrication process of hollow- tube scaffolds for vascularized bone regeneration using coaxial 3D- printing [89]. Reprinted with permission from Zhang et al., Copyright © 2024 Elsevier Ltd. (**D**) Schematic illustration of SHED-derived hypoxic exosome generation process and their application in the rat calvarial de-fect model for vascularized bone regeneration [106]. Reprinted with permission from Gao et al., Copyright © 2024 Elsevier Ltd. (**E**) Schematic illustration of the structural design and fabrication process of a 3D-printed vascularized bone construct using GelMA hydrogel, HUVECs, and MSCs [107]. Reprinted with permission from Cui et al., Copyright © 2024 John Wiley & Sons, Inc.

The administration of angiogenic growth factors is typically used in bone regeneration to promote the formation of new bone tissue and blood vessels. Commonly used growth factors, such as VEGF and bone morphogenetic protein 2 (BMP-2), play a critical role in stimulating angiogenesis and osteogenesis [29,113]. By delivering these growth factors directly to the site of bone defects, it can provide necessary signals to promote bone and blood vessel formation, ultimately aiding in the successful regeneration of bone tissue. In one example, the simultaneous release effects of VEGF and BMP-2 on bone regeneration in rat critical-size defect models were evaluated [85]. The results showed that dual administration of VEGF and BMP-2 significantly increased bone formation and bone bridging compared to BMP-2 alone or no growth factor at both 4 and 12 weeks [85]. Additionally, the dual group had a higher percentage of blood vessel volume within the defect at 4 weeks compared to the other groups in microCT imaging results, indicating enhanced angiogenesis [85]. These findings suggest that delivering an angiogenic and an osteogenic factor simultaneously provides a synergistic response that would promote bone regeneration in critical-size defects [85]. Another study investigated the potential of a VEGF-coated scaffold to induce vascularization for bone regeneration [37]. VEGFs were released from the scaffolds in a sustained manner for more than 14 days, and microCT imaging results at 12 weeks showed near-complete bridging of the bone defect by the newly formed mineralized tissue in the coated VEGF-releasing scaffolds [37]. A quantitative analysis showed an increase in bone volume fraction and bone mineral density in the coated VEGF-releasing scaffolds compared to the control scaffolds [37]. In conclusion, the study demonstrated the angiogenic capacity of growth factor-coated scaffolds and their potential to induce vascularization, which is critical for bone formation and healing [37]. However, the osteogenic effect was not as significant, probably due to the low concentration of scaffold material that was used in the study [37]. A different study demonstrated the efficacy of administering angiogenin in vascularized bone regeneration [86]. In vitro cell culture studies showed that angiogenin-loaded scaffolds enhanced cell proliferation and adhesion, indicating that angiogenin stimulates EC proliferation and angiogenesis [86]. In addition, the study found that angiogenin enhanced the adhesion of ECs to the scaffold, suggesting enhanced bone formation through angiogenesis [86]. The in vivo rabbit calvarial defect model demonstrated that angiogenin-containing scaffolds induced an increased number of blood vessels with increasing angiogenin concentrations, and also showed an extensive neo-bone structure around the defect area that fused with the host’s bone 8 weeks post-implantation (Figure 2A) [86]. Collectively, the results indicate that angiogenin promotes angiogenesis and bone formation, suggesting that it is a potential growth factor for bone tissue engineering applications [86]. Another example used a polyhedral oligomeric silsesquioxane (POSS)-modified gelatin hydrogel to promote vascularization in bone regeneration (Figure 3A) [87]. Compared to 0% POSS hydrogels, the 3% POSS hydrogel coupled with VEGF/BMP-2 demonstrated a significantly higher degree of vascularization in microCT imaging and CD31 immunohistochemical staining [87]. In addition, the 3% POSS hydrogel coupled with VEGF/BMP-2 showed enhanced bone regeneration in the in vivo rat calvarial defect model, with complete healing of the defect and no gaps observed, as well as the largest number of newly formed blood vessels, indicating effective bone repair and angiogenesis effects (Figure 2B) [87]. Morphometric analysis results showed that the 3% POSS hydrogel coupled with VEGF/BMP-2 also had the highest bone mineral density, bone volume to total volume, and trabecular number [87]. This suggests that the POSS-modified hydrogel is highly effective in promoting vascularized bone repair [87]. In a different study, whitlockite (WH) nanoparticles and VEGF were utilized to show how the combined microenvironment of these two substances synergistically enhanced the osteogenesis and angiogenesis of MSCs [88]. As a result, enhanced osteogenic differentiation and vascularization were observed in the WH and VEGF scaffold in vitro [88]. In a rat calvarial defect model, the WH and VEGF synergistic group showed the most significant effect in promoting bone regeneration and blood vessel formation at the defect site [88]. In another study, 3D-printed scaffolds with a hollow tube structure and bioactive ions, referred to as BRT-H scaffolds, were fabricated using a coaxial 3D-printing technique (Figure 3B,C) [89]. The hollow tube microstructure and the release of bioactive ions synergistically enhanced angiogenesis and osteogenesis, as evidenced by the stimulation of EC migration and blood vessel formation [89]. The BRT-H scaffolds exhibited high compressive strength and facilitated blood vessel ingrowth and an enhanced delivery of stem cells and growth factors [89]. In a rabbit radial defect model, the scaffolds promoted early angiogenesis and subsequent bone regeneration compared to the control group, evidenced by the microCT image of enhanced blood vessel formation (Figure 2C) [89]. The study showed that the coaxial printing of hollow-tube-structured BRT-H scaffolds was effective in promoting vascularized bone regeneration in large segmental bone defects [89]. Another study developed a nanofibrous scaffold with interconnected microchannels for mimicking the bone microenvironment and facilitated the sequential release of the pro-angiogenic drug dimethyloxalylglycine [90]. In vitro results showed an increased expression of angiogenic and osteogenic genes, increased alkaline phosphatase (ALP) activity, and increased mineral deposition, indicating the potential of the scaffold to stimulate both angiogenesis and osteogenesis [90]. In vivo, the scaffold significantly promoted vascularization and bone regeneration in a rat calvarial defect model, suggesting that the interconnected microchannel structures successfully supported an effective drug delivery system within the scaffold for enhanced vascularization and bone regeneration (Figure 2D) [90]. A different example developed a 3D-bioprinted scaffold composed of silk and hydroxyapatite loaded with BMP-2, VEGF, and NGF to enhance angiogenesis, osteoblast differentiation, and bone regeneration [91]. The results showed that the 3D-bioprinted bone constructs were cytocompatible and osteoconductive, as evidenced by the enhanced osteogenic differentiation of MSCs. In particular, the incorporation of VEGF within the construct was effective in enhancing HUVEC migration, thereby improving the vascularization potential for bone repair and regeneration [91].

### 4.2. Cell Delivery

Conventional approaches to bone regeneration rely on a single cell type to promote bone formation. Yet, these methods fail to encapsulate the intricacies inherent in the complex processes of bone regeneration, where osteogenesis and angiogenesis engage in a harmonious interplay. In particular, angiogenesis is essential for osteogenesis to ensure an adequate supply of nutrients and oxygen [114]. Some cases have highlighted the significance of co-culturing different cell types, demonstrating their pivotal role in both osteogenesis and angiogenesis. Most commonly, it is known that ECs and mesenchymal stem cells (MSCs) work in concert to increase the efficiency of osteogenesis, which speeds up the vascularization process in bone regeneration. One example performed a co-transplantation of ECs and bone marrow stromal cells (BMSCs) on biodegradable polymer scaffolds to investigate whether Ecs could directly influence the osteogenic potential of BMSCs [92]. When BMSCs were co-cultured with Ecs, an early osteogenic marker (ALP expression) and late osteogenic marker (osteocalcin) greatly increased compared to the culture of BMSCs alone. In addition, the blood vessels were examined using CD31 immunostaining to identify functional vessels to determine if transplanted the ECs improved neovascularization [92]. The proportion of transplanted EC-derived vessels among the total number of vessels showed a significant increase in scaffolds co-cultured with BMSCs and ECs compared to scaffolds with BMSCs alone [92]. However, the overall number of vessels generated within the scaffolds throughout the 8-week observation period did not differ statistically significantly between these two groups [92]. Another study investigated the bone regeneration effects of incorporating ECs along with osteoblasts into the PCL-HA composite (Figure 2E) [93]. In vivo experiments showed that scaffolds seeded with ECs and osteoblasts exhibited enhanced vascularization and osteogenesis, as well as improved mechanical properties of the engineered bone tissue compared to scaffolds containing only osteoblasts (Figure 2E) [93]. Three-dimensional microCT imaging results further supported the efficacy of the EC–osteoblast co-culture group in promoting bone defect repair compared to the osteoblast-only group, highlighting the significant contribution of ECs in the repair process [93]. Overall, these results suggest that the co-culture of ECs with osteoblasts has potential for clinical use in the treatment of large bone defects [93]. A different research study used a co-culture approach between MSCs and MSC-derived ECs in a porous β-tricalcium phosphate scaffold for repairing large segmental bone defects in rabbits [94]. The results showed that the co-culture group containing ECs promoted the osteogenic effect of MSCs with improved mechanical properties and a more abundant capillary network, suggesting that it is an effective approach to enhance osteogenesis and angiogenesis in bone regeneration [94].

### 4.3. Gene Delivery

The gene delivery method is a pivotal component in bone regeneration, as it facilitates the targeted delivery and expression of angiogenic or osteogenic genes within the defect site. This process leads to the production of vessel-forming or bone-inducing proteins, significantly enhancing the bone-repairing process. Through the use of gene-activated matrices and other delivery platforms, sustained release and localized expression of these genes can be achieved, providing a controlled environment for bone tissue formation and vascularization. In one study, researchers developed a bioactive scaffold for gene delivery to enhance bone repair and vascularization [95]. The scaffold was designed to deliver plasmid DNA encoding for VEGF and BMP-2 using non-viral vectors [95]. The in vitro results demonstrated that the dual delivery scaffold made of polyethylenimine and nano-hydroxyapatite significantly enhanced calcium deposition and mineralization [95]. In vivo, the gene-activated scaffolds recruited and transfected host cells, as demonstrated by the presence of GFP-expressing cells in a rat calvarial defect model. This method effectively enhanced vessel formation in the bone defect region by providing a localized and sustained delivery of the angiogenic gene (VEGF) [95]. In another paper, the researchers delivered the VEGF gene to enhance angiogenesis and bone regeneration [96]. They found that VEGF gene delivery, particularly through a gene-activated matrix, effectively bridged bone gaps and increased vessel formation in a rabbit radial defect model [96]. The utilization of a collagen sponge as a delivery system for the non-viral VEGF gene application resulted in increased angiogenesis and osteoid formation, as confirmed through a radiographic evaluation, μCT scans, histology, and immunohistochemical staining for blood vessels [96]. This approach was shown to be particularly effective in promoting vascularization and bone regeneration in large segmental defects, suggesting its potential as a valuable tool for treating nonunion [96]. Another study investigated the use of angiopoietin 1 (Ang1) gene-transfected MSCs seeded onto beta-tricalcium phosphate (β-TCP) scaffolds for repairing segmental bone defects in rabbits [97]. The results showed that the experimental group receiving the Ang1-transfected MSCs exhibited increased vessel growth, faster bone union, and higher bone formation compared to the control group, resulting in successful repair of the segmental bone defect within 12 weeks [97]. The study showed that the experimental group’s expression of Ang1 significantly improved angiogenesis by promoting capillary regeneration within the porous network [97]. In a different study, the proangiogenic gene miR-21 and the pro-osteogenic gene miR-5106 were delivered to the site of the bone defect using zeolitic imidazolate framework 8 as a non-viral vector for promoting angiogenesis and bone regeneration [98]. The in vivo experiments, using a rat calvarial defect model, demonstrated significant improvements in blood vessel formation, bone mineral density, trabecular number, and thickness in the experimental group [98]. The success of the method in enhancing angiogenesis for bone regeneration was attributed to the efficient delivery and release of therapeutic miRNAs within cells, facilitated by the miRNA delivery nanocomposites [98]. This was demonstrated through an in vitro scratch assay and tube formation assay, which showed the enhanced angiogenic ability of the gene-incorporated nanocomposites [98].

### 4.4. Perfusable 3D Vascular Network

Mimicking a perfusable 3D vascular network is crucial in osteogenesis and angiogenesis since it closely replicates the natural environment of bone tissue, which is essential for transporting nutrients, oxygen, and waste removal, as well as providing mechanical cues to cells [48]. A perfusable 3D vascular network within a scaffold ensures that these critical processes can occur throughout the entire construct, thereby supporting the growth of viable tissue constructs. In addition, the dynamic fluid flow within the vascular network can simulate the physiological shear stress that cells experience in the in vivo environment. In one study, a nanocoating system was integrated with a biomimetic, 3D-bioprinted, perfused microstructure to create a vascularized bone complex [99]. This construct was designed to mimic the hierarchical architecture of native bone and to provide a dynamic environment for cell growth, with a focus on regulating angiogenesis and osteogenesis through a matrix metalloprotease 2-responsive mechanism [99]. The perfused microstructure system was critical in simulating in vivo conditions and allowing for the dynamic culture of cells [99]. The results demonstrated that the nanocoating-modified 3D-bioprinted scaffolds had great bioactivity and the potential to form a vascularized bone complex [99]. In a different study, 3D-bioprinted scaffolds with perfusable vascular channels were created to enhance bone regeneration by promoting osteogenesis and angiogenesis [100]. The bioprinted scaffolds significantly promoted the osteogenic differentiation of MSCs, and the presence of human umbilical vein endothelial cells (HUVECs) within the scaffolds resulted in an enhanced formation of vascular networks, particularly under dynamic culture conditions that mimic the in vivo environment [100]. The use of GelMA hydrogel bioink was also instrumental in forming these vascular networks and providing the necessary mechanical robustness within the scaffold [100]. This approach effectively enhanced angiogenesis in bone regeneration by creating a conducive environment for vascularization through the strategic use of bioink formulations, co-culture systems, and 3D-printing techniques together [100].

### 4.5. Hydrogels Inducing Angiogenesis

Hydrogel-based vascularized bone models mimic the intricate function of the natural bone extracellular matrix (ECM). These hydrogel platforms can be precisely patterned or modified to encapsulate different cell types and loaded with different particles to promote bone regeneration. The ability to engineer constructs with spatially organized vascular networks and osteogenic niches hold promise for the treatment of complex bone defects, offering a more tailored and potentially effective healing strategy compared to traditional bone repair methods. In one study, researchers engineered a hydrogel-based vascularized bone model that demonstrated the effective formation of mineralized areas surrounded by vasculature, crucial for bone tissue engineering [101]. The existence of osteogenic and angiogenic niches within the model resulted in enhanced bone formation, with the angiogenic niche significantly improving this regeneration [101]. The hydrogel platform, which included endothelial and mesenchymal stem cells, exhibited self-assembly into cord-like structures, indicating the potential for these engineered tissues to connect with the host’s vasculature upon implantation and support cell survival and function [101]. In another study, a hydrogel-microsphere system was developed for the delivery of integrated biological signal peptides to enhance bone repair [102]. The synthesized composite microspheres, particularly the gelatin methacrylate (GelMA)-S-B microspheres, demonstrated good biocompatibility, sustained osteogenic capacity, and preserved vascular properties [102]. When tested in a rat femoral defect model, the microspheres effectively promoted bone formation, with histologic evaluations confirming increased new bone formation in the GelMA-S-B group compared to controls [102]. These results indicate that the hydrogel-microsphere system is an effective platform for delivering biological signals that can significantly enhance bone regeneration, with promising potential for clinical applications in bone repair and tissue engineering [102]. In another example, they developed a bilayer hydrogel designed to achieve vascularized bone regeneration in bone defects [103]. The hydrogel platform achieved vascularization within the bone structure by simultaneously incorporating magnesium-modified black phosphorus nanosheets to promote angiogenesis and β-tricalcium phosphate nanocrystals to promote osteogenic differentiation [103]. The magnesium ions in the nanosheets promoted angiogenesis and greatly enhanced the in vitro adhesion and proliferation of ECs [103]. The in vivo results indicated that the hydrogel scaffold facilitated bone healing in rat calvarial defect models based on increased bone mineral density and bone score values, with a complete coverage of the defect site with newly formed bone at 12 weeks after hydrogel bilayer transplantation [103]. These results suggest that the hydrogel bilayer scaffold is effective in promoting bone regeneration, with the added benefit of promoting angiogenesis [103]. In a different study, a biomimetic periosteum consisting of a diselenide-containing gelatin and calcium alginate (Gel-Se/Alg-Ca) hydrogel was developed for effective bone regeneration [104]. The biomimetic periosteum was effective in promoting bone regeneration through the continuous release of nitric oxide (NO), which activated the NO-cGMP signaling pathway, thereby enhancing both osteogenesis and angiogenesis [104]. The in vivo results in a rat calvarial defect model showed that the Gel-Se/Alg-Ca group exhibited the most efficient bone tissue repair effect, with a higher bone volume fraction and bone mineral density compared to other groups [104].

### 4.6. Extracellular Vesicle (EV) Delivery

Extracellular vesicles (EVs) are released by living cells, including mammalian cells and microorganisms, and consist of membranous and intracellular components. EVs play a critical role in intercellular communication, making them important in regenerative medicine applications. In contrast to the challenges of common cell delivery methods, such as unpredictable cell fates and entrapment in pulmonary capillaries, exosomes, although non-living, carry biological information, evade entrapment, and can be cryopreserved for efficient storage and later use. In one study, bone marrow-derived EVs were preconditioned under hypoxia to enhance bone regeneration [105]. These EVs were effectively delivered using an injectable bioactive polypeptide-based hydrogel, which not only demonstrated good biocompatibility and a sustained release of EVs, but also resulted in excellent bone regeneration in a rat calvarial defect model [105]. The study also highlighted that hypoxic EVs were superior at promoting vascularization, osteoblast proliferation, migration, and differentiation compared to EVs from normoxic conditions [105]. Another study used exosomes derived from the stem cells of human exfoliated deciduous teeth (SHEDs) to investigate their potential in bone regeneration (Figure 3D) [106]. The SHEDs preconditioned under hypoxic conditions produced exosomes with an enhanced ability to promote angiogenesis and osteogenesis [106]. These SHED-derived hypoxic exosomes were then successfully loaded onto poly(lactic-co-glycolic acid) microspheres coated with polydopamine to facilitate sustained release [106]. In vivo experiments using a rat calvarial defect model demonstrated that the microsphere delivery of exosomes significantly promoted new bone formation and vascularization [106]. High-throughput RNA sequencing indicated that these preconditioned exosomes could regulate pathways related to bone tissue regeneration, and the released exosomes maintained their biological functions, effectively enhancing angiogenesis and bone regeneration [106].

### 4.7. Three-Dimensional-Bioprinted Models

Three-dimensional-bioprinted vascularized bone models enable the precise fabrication of bone tissue with integrated vascular networks through advanced 3D-bioprinting techniques that allow for the simultaneous deposition of cells and biomaterials in a spatially controlled manner. By incorporating bioactive factors such as growth factors into the constructs, these models can also provide the targeted stimulation of both osteogenesis and angiogenesis at specific regions within the tissue, and the combination of these factors within the 3D-bioprinted constructs can lead to improved therapeutic outcomes for bone regeneration. In addition, the dynamic culture of these 3D-bioprinted constructs allows for better nutrient exchange, which is beneficial for the maturation of the vascular network and overall bone regeneration [115,116]. In one study, 3D-bioprinting technology was used to create vascularized bone constructs using GelMA hydrogel, HUVECs, and MSCs (Figure 3E) [107]. The incorporation of MSCs was found to stabilize the endothelial tubes, resulting in improved cell adhesion and proliferation [107]. The bioprinted construct showed an increased secretion of VEGFs and collagen I content, along with enhanced osteogenic differentiation due to the presence of bioactive peptides within the construct [107]. Using a custom-designed flow bioreactor system, the authors were able to dynamically culture the constructs, resulting in extensive capillary networks and increased osteogenic and angiogenic differentiation [107]. The results highlight the potential of this novel 3D-bioprinting approach, combined with regional bioactive peptide immobilization, to produce complex, vascularized bone constructs with significant therapeutic efficacy for bone regeneration [107]. Another example presented a 3D-bioprinted scaffold loaded with deferoxamine (DFO) liposomes to enhance bone regeneration through osteogenic and angiogenic properties [108]. DFO is known to promote both osteogenesis and angiogenesis by inducing the expression of hypoxia-inducible factor 1-alpha and VEGF [108]. The scaffold promoted osteogenesis in BMSCs and upregulated osteogenic-related gene and protein expression [108]. In vivo experiments using a rat femoral defect model demonstrated new bone growth and enhanced angiogenesis due to the controlled release of DFO [108]. This release mechanism stimulated the early-stage internal vascularization and maturation of the vascular network, which is critical for coupling angiogenesis with osteogenesis and ultimately promoting bone regeneration [108]. In a different research study, they developed and evaluated gelatin-based bioinks for an extrusion-based method to create bioprinted vascularized bone equivalents [109]. They modified gelatin hydrogel formulation to enhance 3D vascularization and successfully bioprinted co-culture hydrogels containing human dermal microvascular endothelial cells (HDMECs) and adipose-derived stem cells (ASCs) [109]. The resulting hydrogels demonstrated an enhanced ability to form capillary-mimicking structures and supported the deposition of bone-specific proteins [109]. This method effectively enhanced angiogenesis, which is critical for bone regeneration, by providing a conducive environment for angiogenesis and the development of vasculature within the engineered tissue constructs [109].

### 4.8. Other Synthetic Models

Among various other synthetic models, microfluidic vascularized bone models are recently emerging in vitro platforms that integrate microfluidic technology with 3D tissue engineering to create a controlled environment for studying the interactions between bone cells and blood vessels. By incorporating materials such as hydroxyapatite into the ECM materials, these models can mimic the mineralized matrix of natural bone while also allowing paracrine communication between cells [117]. While most bone-on-a-chip models cannot mimic the vasculature in the system, microfluidic vascularized bone models can closely resemble the dynamic in vivo microenvironment. One example demonstrated a microfluidic vascularized bone model with the integration of hydroxyapatite (HA) and a fibrin ECM, resulting in enhanced angiogenic properties [110]. The presence of HA improved angiogenic characteristics such as the sprout length, velocity, number, and lumen diameter, indicating a more robust and dynamic formation of vascular networks [110]. The microfluidic device enabled paracrine communication between ECs and stromal cells during vessel formation, which is critical for mimicking in vivo bone angiogenesis [110]. In addition, the biocompatibility of HA with the microvascular endothelium was confirmed, as it maintained healthy endothelial markers and supported the expression of functional endothelial markers that contribute to angiogenesis [110]. In particular, this experiment did not include additional in vivo studies, but microfluidic models have great potential for in vivo applications, as shown in other examples of successful transplantation of vascularized microfluidic models [118]. In addition to microfluidic models, other synthetic models have used innovative methods to induce angiogenesis in bone regeneration. A different study developed a biomimetic membrane with HA nanoparticle micropatterns to mimic the natural periosteum and promote bone regeneration [111]. These membranes showed efficient cell adhesion and successfully induced the cell alignment and differentiation in vitro, and MSCs cultured on the membranes showed greater osteogenesis and angiogenesis [111]. The in vivo experiments in a rat calvarial defect model demonstrated that the biomimetic membranes not only enhanced bone regeneration but also significantly promoted blood vessel formation and ossification at the defect site by providing a conducive microenvironment for cell orientation and the sustained release of growth factors from calcium phosphate, which are key to effective vascularization and bone tissue regeneration [111]. Another study investigated the effects of a static magnetic field and magnetic scaffolds on osteoblast differentiation, bone formation, and angiogenesis [112]. Their results showed that the application of a magnetic field in combination with magnetic scaffolds significantly stimulated osteoblast functions, resulting in enhanced bone regeneration [112]. This combination was also found to promote VEGF expression and capillary tube formation [112]. When implanted in a mouse calvarial defect model for 6 weeks, the magnetic scaffold filled the defect gap more densely with newly generated bone tissue and showed a highly calcified structure, suggesting that the use of a magnetic field may be a potential tool for inducing angiogenesis in bone tissue regeneration [112].

## 5. Conclusions and Future Perspectives

In this review, we provide an overview of the angiogenic strategies that promote bone regeneration. Since bone, as a highly vascularized tissue, undergoes hypoxia due to bleeding in bone fractures, creating new vasculatures connected with host blood vessels has emerged as a promising strategy for bone tissue engineering. The neovasculature in the bone defect provides oxygen and the required nutrients necessary for osteogenesis, and also secretes angiogenic factors that stimulate osteogenesis. Conventional tissue engineering indirectly induces bone angiogenesis by fabricating porous scaffolds that lead to EC migration due to the hypoxic condition inside the scaffold. Meanwhile, recent strategies reviewed in this paper focus on the direct induction of angiogenesis by releasing proangiogenic proteins, genes, cells, or EVs, as well as the incorporation of specific mechanical and structural properties. It is important to note that each approach has its own advantages and disadvantages, which can impact therapeutic efficacy and safety. For instance, angiogenic growth factors such as VEGF are a primary means of inducing angiogenesis, while their application raises concerns about the potential for inducing aberrant physiologies, including leaky vasculature or tumorigenesis, particularly at high dosages [119]. Cell therapy and gene therapy present alternative strategies to growth factors, yet their long-term safety remains inadequately evaluated, and their therapeutic efficacy has not demonstrated superiority over VEGF treatment. EV treatment is emerging as a potential angiogenic strategy with its high safety and therapeutic efficacy, but is hindered by low productivity and high costs, which pose challenges for clinical translation. Scaffold engineering represents another avenue for future advancement in bone angiogenesis without the need for angiogenic factors, such as the NO-releasing hydrogel or 3D microchannel structures demonstrating the ability to recruit ECs and induce angiogenesis [120]. Nevertheless, the therapeutic effectiveness of mechanical and structural cues still appears to be comparatively insufficient compared to biochemical treatments. We further expect the integration of nanostructures to be a transformative force in bone regeneration, as well as microfluidic devices that replicate perfusable vascularized bone as potential implantable devices, which are currently being explored with better regenerative efficacy. Overall, ongoing research into the development and application of angiogenesis in bone regeneration holds tremendous potential for advancing the relevant fields and establishing novel strategies for targeted and efficient therapeutic interventions.

## Figures and Tables

**Table 1:** A summary of vascularization/angiogenic strategies for bone regeneration.

Strategy	Main Factors	Findings and Observations	Ref.
Angiogenic factor delivery	VEGF, BMP-2	Vessel formation ↑/Bone formation, bone bridging ↑	[85]
	VEGF	Bone bridging, bone volume fraction, bone mineral density ↑	[37]
	Angiogenin	EC proliferation, adhesion ↑/Vessel formation ↑/Bone formation ↑	[86]
	VEGF, BMP-2	Vessel number ↑/Bone formation ↑	[87]
	VEGF	Osteogenic differentiation ↑/Vessel formation ↑/Bone formation ↑	[88]
	Bioactive ions	EC migration ↑/Vessel formation ↑/Stem cell, growth factor delivery ↑/Bone formation ↑	[89]
	Dimethyloxalylglycine	Angiogenic, Osteogenic marker expression ↑/ALP activity ↑/Vessel formation ↑/Bone formation ↑	[90]
	BMP-2, VEGF, NGF	Osteogenic differentiation ↑/HUVEC migration ↑	[91]
Cell delivery	ECs, BMSCs	Osteogenic marker expression ↑/Vessel formation ↑	[92]
	ECs, osteoblasts	Vessel formation ↑/Bone formation ↑	[93]
	MSCs, MSC-derived ECs	Mechanical properties ↑/Capillary network ↑	[94]
Gene delivery	VEGF, BMP-2 gene	Calcium deposition, mineralization ↑/Host cell recruitment, transfection ↑/Vessel formation ↑	[95]
	VEGF gene	Bone gap ↓/Vessel formation ↑ Osteoid formation ↑	[96]
	Ang1 gene	Vessel growth ↑/Faster bone union/Bone formation ↑	[97]
	miR-21, miR-5106	Vessel formation ↑/Bone mineral density, trabecular number, thickness ↑/Angiogenic ability ↑	[98]
Perfusable 3D vascular network	Perfused microstructure	Osteogenic differentiation ↑/Vascular like network ↑	[99]
	Perfusable vascular channels	Osteogenic differentiation ↑/Vessel formation ↑	[100]
Angiogenesis-inducing hydrogel	osteogenic and angiogenic niche-incorporated hydrogel	EC, MSC self-assembly/Bone formation ↑	[101]
	Biological signal peptide	Bone formation ↑/Vascularization ↑	[102]
	Bilayer hydrogel	EC proliferation, adhesion ↑/Bone mineral density, bone score values, bone formation ↑/Angiogenesis ↑	[103]
	NO-releasing biomimetic periosteum	Angiogenesis ↑/Bone volume fraction, bone mineral density ↑	[104]
EV delivery	Bone marrow-derived hypoxic EVs	Vascularization ↑/Osteoblast proliferation, migration and differentiation ↑/Bone formation ↑	[105]
	SHED-derived hypoxic exosomes	Vascularization ↑/Bone formation ↑	[106]
3D-bioprinting	HUVECs, MSCs, GelMA hydrogel	Cell adhesion, proliferation ↑/VEGF, collagen I ↑/Osteogenic, angiogenic differentiation ↑/Capillary network ↑	[107]
	DFO liposome-containing scaffold	Osteogenic marker expression ↑/Bone growth ↑/Early-stage internal vascularization ↑/Vascular network maturation ↑	[108]
	HDMECs, ASCs, gelatin-based bioink	Capillary-mimicking structure formation ↑/Bone-specific protein ↑/Vascularization ↑	[109]
Other synthetic methods	HA-incorporated microfluidic model	Vessel sprout length, velocity, number, and lumen diameter ↑/ECs–stromal cells paracrine communication/Functional endothelial marker expression ↑	[110]
	Biomimetic membrane with HA nanoparticle	Cell adhesion, alignment, differentiation ↑/Vessel formation ↑/Ossification ↑	[111]
	Static magnetic field, magnetic scaffold	Osteoblast function ↑/VEGF expression ↑/Capillary tube formation ↑/Bone formation ↑	[112]

↑, indicating an increase; ↓, indicating a decrease; VEGF, vascular endothelial growth factor; BMP-2, bone morphogenetic protein 2; EC, endothelial cell; ALP, alkaline phosphatase; NGF, neural growth factor; HUVEC, human umbilical vein endothelial cell; BMSC, bone marrow stromal cell; MSC, mesenchymal stem cell; Ang1, angiopoietin 1; EV, extracellular vesicle; DFO, deferoxamine; HDMEC, human dermal microvascular endothelial cell; ASC, adipose-derived stem cell; HA, hydroxyapatite.
